# Cumulative incidence and mortality of infective endocarditis in Siriraj hospital–Thailand: a 10-year retrospective study

**DOI:** 10.1186/s12879-019-4689-5

**Published:** 2019-12-18

**Authors:** Taksaon Angsutararux, Nasikarn Angkasekwinai

**Affiliations:** Division of Infectious Diseases and Tropical Medicine, Department of Medicine, Faculty of Medicine Siriraj Hospital, Mahidol University, 2 Wanglang Road, Bangkoknoi, Bangkok, 10700 Thailand

**Keywords:** Infective endocarditis, IE, Native valve IE, Prosthetic valve IE

## Abstract

**Background:**

To investigate the cumulative incidence of and factors associated with mortality among patients with infective endocarditis (IE) at Thailand’s largest national tertiary referral center.

**Methods:**

Medical charts of adult patients diagnosed with IE by Duke criteria at Siriraj Hospital during January 2005 to May 2015 were retrospectively reviewed.

**Results:**

Of 380 patients, 66.3% had definite IE, and 81.3% had native valve IE (NVE). Cumulative IE incidence was 5.67/1000 admissions. The most common pathogens were viridans group streptococci (VGS) (39.7%), methicillin-sensitive *Staphylococcus aureus* (MSSA) (13.1%), and beta-hemolytic streptococci (11.5%) in NVE; and, MSSA (20.3%), VGS (20.3%), and *Enterococcus* spp. (16.9%) in prosthetic valve (PVE) or device-related IE (DRIE). Overall in-hospital mortality was 18.4%. Mortality was significantly higher in PVE/DRIE than in NVE (26.8% vs. 16.5%, *p* = 0.047). End-stage renal disease (ESRD) (aOR: 9.43, 95% CI: 2.36–37.70), diabetes mellitus (DM) (aOR: 2.81, 95% CI: 1.06–7.49), neurological complication (aOR: 14.16, 95% CI: 5.11–39.22), congestive heart failure (aOR: 4.32, 95% CI: 1.91–9.75), hospital-acquired infection (aOR: 3.78, 95% CI: 1.66–8.57), renal complication (aOR: 3.12, 95%CI: 1.32–7.37), and other complication during admission (aOR: 3.28, 95% CI: 1.41–7.61) were independently associated with mortality.

**Conclusions:**

The incidence of IE, and the mortality rate among those diagnosed with IE are both increasing in Thailand – particularly among those with PVE or DRIE. End-stage renal disease, diabetes mellitus, and development of IE-related complications during admission were found to be independent predictors of mortality.

## Background

Infective endocarditis (IE) poses a great therapeutic challenge due to difficulties in diagnosis and the high risk of morbidity. Since a decrease in the late 1960’s, the in-hospital mortality rate has remained relatively stable at approximately 20% [1]. Within the last decade, the epidemiology of IE has changed significantly worldwide. Recent study found an increase in IE among elderly population with comorbidities, and among patients that use invasive medical devices, such as intracardiac device, prosthetic heart valve, or hemodialysis catheter. These factors were also reported to be at least partially responsible for changing the patterns of the causative pathogens [2, 3]. Several studies reported an increased proportion of staphylococcal IE, and a decreased proportion of IE caused by viridans group streptococci (VGS) [4]. However, most studies in IE were conducted in North America or Europe, and data specific to the epidemiology of IE in Southeast Asia is relatively scarce. Two previous studies were conducted in Northeastern Thailand [5, 6]. The epidemiology of IE may vary among continents, geographic regions, and by type of hospital. Improved understanding of the epidemiology of IE, including the pathogens that cause IE, will help to guide the selection of empirical antibiotic therapy.

The aim of this study was to determine the cumulative incidence, clinical characteristics, microbial etiology, mortality, and factors associated with in-hospital mortality among patients with infective endocarditis admitted to the largest tertiary medical center in Thailand.

## Methods

This retrospective cohort study was conducted at the Division of Infectious Diseases and Tropical Medicine, Department of Medicine, Faculty of Medicine Siriraj Hospital, Mahidol University, Bangkok, Thailand. Siriraj Hospital is the largest tertiary and quaternary-care medical center in Thailand, with a capacity of more than 2000 beds and *more than* one million outpatient visits per year. All adult patients aged over 15 years who were admitted during 1 January 2005 to 31 May 2015 that were diagnosed with infective endocarditis (IE) according to modified Duke criteria were included. Diagnosis of IE was identified from the computer record using ICD-10 I33.0 as the diagnostic code. Patient data relating to demographics, clinical characteristics, microbiological findings, occurrence of complication, echocardiographic findings, treatment, and outcome were collected, recorded, and analyzed.

The presumed mode of acquisition of IE was categorized as community-acquired IE (CA-IE) or healthcare-associated IE (HA-IE). HA-IE was further subdivided into nosocomial or non-nosocomial. Nosocomial IE (NIE) was defined as IE that developed in a patient hospitalized > 48 h prior to the onset of signs/symptoms consistent with IE. Non-nosocomial IE (NNIE) was defined as signs and/or symptoms of IE starting < 48 h after admission in a patient with healthcare contact consisting of home-based nursing or intravenous therapy, hemodialysis or intravenous chemotherapy < 30 days, hospitalized in an acute care facility < 90 days before the onset of IE, or resident in a nursing home/long-term care facility [7]. All cases not fulfilling the criteria for healthcare-associated infection were defined as community-acquired IE. The type of heart involvement was classified as native valve IE (NVE), prosthetic valve IE (PVE), or device-related IE (DRIE). DRIE was defined as IE developing on pacemaker or defibrillator wires with or without associated valve involvement. Abnormal echocardiogram was defined as vegetation, abscess, or new dehiscence of a prosthetic valve from transthoracic echocardiography (TTE) or transesophageal echocardiography (TEE) [7].

Inappropriate antibiotic was defined as a prescription for an antibiotic to which the organism was non-susceptible in vitro according to the identified organism from hemoculture or tissue culture, as well as antibiotic other than guideline recommendation [7] in culture-negative IE. The primary outcome was in-hospital mortality.

### Sample size calculation

Previous study reported the incidence of in-hospital mortality rate among patients with IE to be 14% [8]. The sample size was a priori calculated to estimate a single proportion with precision of 3.5% and a confidence interval of 95% using n Query Advisor program (Statistical Solutions, Ltd., Cork, Ireland). Therefore, the sample size needed to determine incidence of in-hospital mortality was 378 participants.

We performed post-hoc power analysis using G*Power 3.1.9.2 after a study has been completed to investigate a type II error in the study. A logistic regression of mortality on neurological complication at diagnosis as one covariate and adjusted for the other covariates in the model with a sample size of 308 participants (of which 17.6% were in the neurological complication group and 82.4% were in the non-neurological complication group) achieves 60% power for a two-sided test at 0.05 significance level to detect a change in probability of mortality from the baseline value of 0.152 to 0.286. This change corresponds to an odds ratio of 2.24. An adjustment was made since a multiple logistic regression of the independent variable with the largest *p*-value (neurological at diagnosis) on the other independent variables in the logistic regression model obtained an R-Squared of 0.06.

### Statistical analysis

Data analysis was performed using SPSS Statistics software version 23 for Windows (SPSS, Inc., Chicago, IL, USA). Descriptive statistics were used to summarize patient demographic and baseline clinical characteristics. Categorical variables, including mortality rate, were reported as frequency and percentage. The cumulative incidence of IE referred to the proportion of IE among patients admitted to the hospital and was reported as number of patients with IE per 1000 medical admissions. Continuous data are expressed as mean plus/minus standard deviation (SD) or median and range, as appropriate. Chi-square test or Fisher’s exact test was used to analyze for association between categorical clinical variables. Independent samples *t*-test or Mann-Whitney U test was used to analyze for association between a continuous variable and a categorical variable with two categories. Variables with a *p*-value < 0.05 were further analyzed for independent association with mortality using multiple logistic regression. Those results are reported as adjusted odds ratio (aOR) and 95% confidence interval (95% CI). A *p*-value of < 0.05 was considered statistically significant for all tests.

## Results

A total of 380 patients were enrolled in this study for a cumulative incidence of IE of 5.67 per 1000 admissions. Of those, 318 patients (83.7%) were diagnosed IE at the time of admission and were admitted to a medical ward, and 62 patients (16.3%) were transferred or referred for surgery and admitted to the cardiovascular and thoracic (CVT) surgery ward. The clinical characteristics of the 380 included patients are shown in Table 1. The median age of all patients was 53 years (range: 15–91), and most patients (63.4%) were male. Half of all patients had at least one comorbid disease. Among the 380 patients, 252 (66.3%) had definite IE, and 128 (33.7%) were classified as possible IE. Three hundred and nine patients (81.31%) had native valve endocarditis (NVE), 65 patients (17.01%) had prosthetic valve endocarditis (PVE), and 6 patients (1.58%) had device-related infective endocarditis (DRIE).

**Table 1:** Demographic and clinical characteristics of 380 patients according to type of heart involvement

Characteristics	All IE (*n* = 380) n (%)	NVE (*n* = 309) n (%)	PVE or DRIE (*n* = 71) n (%)	*p*-value
Age (years), median (range)	53 (15–91)	53 (15–91)	59 (16–85)	0.081
Male gender	241 (63.4)	197 (63.8)	44 (62.0)	0.779
Comorbid disease	187 (49.2)	142 (46.0)	45 (63.4)	0.008
Hypertension	91 (23.9)	66 (21.4)	25 (35.2)	0.014
Diabetes	57 (15.0)	42 (13.6)	15 (21.1)	0.109
Hyperlipidemia	38 (10.0)	27 (8.7)	11 (15.5)	0.087
End-stage renal disease	21 (5.5)	17 (5.5)	4 (5.6)	0.965
Cancer	21(5.5)	17 (5.5)	4 (5.6)	0.965
Coronary artery disease	16 (4.2)	7 (2.3)	9 (12.7)	< 0.001
IE characteristic				
Definite IE	252 (66.3)	215 (69.6)	37 (52.1)	0.005
Possible IE	128 (33.7)	94 (30.4)	34 (47.9)	
Mode of acquisition of IE				
Community-acquired	321 (84.5)	280 (90.6)	41 (57.7)	1.00
Nosocomial associated	45 (11.8)	20 (6.5)	25 (35.2)	< 0.001
Non-nosocomial associated	14 (3.7)	9 (2.9)	5 (7.0)	0.015
Abnormal echocardiogram^a^	326/372 (87.6)	278/305 (91.1)	48/67 (71.6)	< 0.001
Affected valve				
Mitral valve	166 (43.7)	152 (49.2)	14 (19.7)	< 0.001
Aortic valve	105 (27.6)	85 (27.5)	20 (28.2)	0.911
Multiple valve	19 (5.0)	16 (5.2)	3 (4.2)	1.000
Tricuspid valve	9 (2.4)	7 (2.3)	2 (2.8)	0.677
Other	26 (6.8)	17 (5.5)	9 (12.7)	0.039
Vegetation observed^b^	239/331 (72.2)	209/269 (77.7)	30/62 (48.4)	< 0.001
Complication at diagnosis	229 (60.3)	186 (60.2)	43 (60.6)	0.954
Congestive heart failure	145 (38.2)	111 (35.9)	34 (47.9)	0.61
Arrhythmia	8 (2.1)	3 (1.0)	5 (7.0)	0.001
Neurological	67 (17.6)	61 (19.7)	6 (8.5)	0.024
Other	37 (11.6)	32 (12.5)	5 (7.9)	0.307
Positive hemoculture^c^	311 (82.1)	252 (81.8)	59 (83.1)	0.80
Causative pathogen				
*Streptococcus* spp.	189 (60.8)	169 (67.1)	20 (33.9)	< 0.001
*Staphylococcus* spp.	69 (22.2)	48 (19.0)	21 (35.6)	0.006
*Enterococcus* spp.	31 (10.0)	21 (8.3)	10 (16.9)	0.047
Gram-negative bacilli	10 (3.2)	7 (2.8)	3 (5.1)	0.408
HACEK	6 (1.9)	4 (1.6)	2 (3.4)	0.319
Other Gram-positive bacteria	5 (1.6)	3 (1.2)	2 (3.4)	0.241
Fungus	1 (0.3)	0 (0.0)	1 (1.7)	0.190

A *p*-value< 0.05 indicates statistical significance

^a^Available data 331 cases; ^b^Available data for echocardiography 372 cases

^c^Missing data 1 cases in native valve group

Native valve 309 cases (81.3%)

The demographic and clinical characteristics of patients according to the type of heart involvement are shown in Table 1. Patients with PVE or DRIE were more likely to have comorbid disease (*p* = 0.008), hypertension (*p* = 0.014), coronary artery disease (*p* < 0.001), nosocomial (*p* < 0.001) acquisition, or non-nosocomial acquisition (*p* = 0.015) than patients with NVE. Echocardiographic data were available in 372 (97.9%) patients, and vegetation was observed significantly more frequently in NVE (77.7% vs. 48.4%, *p* < 0.001). Mitral valve was the most commonly affected valve in NVE (49.2%), whereas aortic valve was most frequently affected in PVE or DRIE (28.2%). Congestive heart failure was the most commonly observed complication among the 60% of patients that had complication on admission.

Data on the clinical presentations of IE were retrieved from the charts of those admitted to medical wards. The common manifestations were fever (88.9%), new onset murmur (56.3%), dyspnea (49.1%), and weight loss (18.2%). Vascular phenomena were found in 27.4% of patients, including major arterial embolism (14.5%), conjunctival hemorrhage (8.2%), Janeway lesions (3.8%), intracranial hemorrhage (2.8%), and septic pulmonary infarction (0.3%). Immunological phenomena were observed in 16% of patients, including glomerulonephritis (5.7%), Osler’s nodes (5.0%), rheumatoid factor positive (4.1%), and Roth spots (3.5%). Among the 298 IE patients who had comprehensive fever-related data, 78 patients (26.2%) experienced a decrease in body temperature within 4 days.

**Fig. 1:**
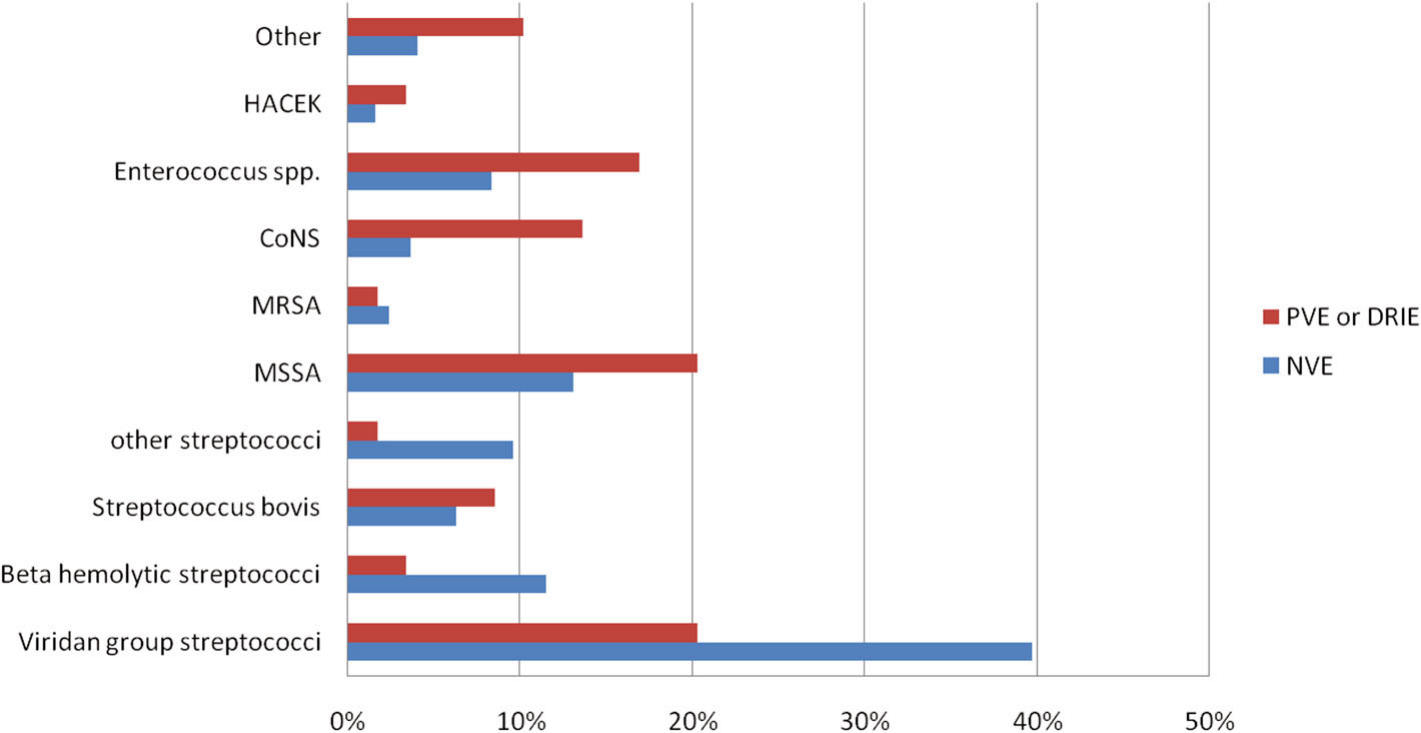
Causative pathogens compared between native valve endocarditis (NVE) and prosthetic valve endocarditis (PVE) or device related endocarditis (DRIE)

A pathogen was identified in 82% of all cases. Overall, *Streptococcus* spp. or *Staphylococcus* spp. was found in the vast majority of IE cases, accounting for 60% and 22%, respectively. The causative pathogens of NVE and PVE or DRIE are shown in Fig. 1. A significant difference was observed in the causative agents causing NVE and PVE or DRIE. VGS (39.7%), MSSA (13.1%), and beta-hemolytic streptococci (11.5%) were the most common pathogens among NVE; whereas, MSSA (20.3%), VGS (20.3%), *Enterococcus* spp. (16.9%), and coagulase-negative staphylococci (CoNS) (13.6%) were more commonly found in PVE or DRIE. The most common type of beta-hemolytic streptococci was *Streptococcus agalactiae* (6.1%).

Seventy of 380 patients died during their hospital stay for an overall in-hospital mortality rate of 18.4%. The mortality rate in the PVE or DRIE group was markedly higher than in the NVE group (26.8% [19 out of 71] vs. 16.5% [51 out of 309], respectively; *p* = 0.047). Univariate analysis for factors associated with mortality is shown in Table 2. The factors that increased mortality in IE were older age (crude OR: 1.04, 95% CI: 1.02–1.05); female gender (crude OR: 1.98, 95% CI: 1.17–3.35); PVE or DRIE group; healthcare-associated IE; presence of comorbidity, including hypertension, diabetes mellitus (DM), hyperlipidemia, or end-stage renal disease (ESRD); infection with non-streptococci; having complication at diagnosis, such as congestive heart failure, arrhythmia, or neurological complications; and, development of complication during admission. Patients who underwent surgical treatment had a lower mortality rate than those receiving only medical treatment (27.1% vs. 54.2%; *p* < 0.001). Patients receiving inappropriate initial antibiotic therapy had a significantly higher in-hospital mortality rate than those who received appropriate empirical antibiotic therapy (25.7% vs. 12.7%, *p* = 0.007). Regarding Gram-negative pathogens, all patients with HACEK IE survived, and only 3 of the 10 patients with Gram-negative non-HACEK IE died.

**Table 2:** Factors associated with overall mortality

Factors	Total (n = 380) n (%)	Alive (*n* = 310) n (%)	Dead (*n* = 70) n (%)	*p*-value
Age (years), mean ± SD		49.5 ± 17.4	59.9 ± 17.8	< 0.001
Male gender	241 (63.4)	206 (66.5)	35 (50.0)	0.011
Type of heart involvement				
Native valve	309 (81.3)	258 (83.2)	51 (72.9)	0.047
PVE + DRIE	71 (18.7)	52 (16.8)	19 (27.1)	
Mode of acquisition of IE				
Community-acquired	321 (84.5)	276 (89.0)	45 (64.3)	
Healthcare-associated	59 (15.5)	34 (11.0)	25 (35.7)	< 0.001
Comorbid disease	187 (49.2)	137 (44.2)	50 (71.4)	< 0.001
Hypertension	91 (23.9)	61 (19.7)	30 (42.9)	< 0.001
Diabetes Mellitus	57 (15.0)	34 (11)	23 (32.9)	< 0.001
Hyperlipidemia	38 (10.0)	26 (8.4)	12 (17.1)	0.031
End-stage renal disease	21 (5.5)	11 (3.5)	10 (14.3)	0.001
Cancer	21 (5.5)	19 (6.1)	2 (2.9)	0.29
Coronary artery disease	16 (4.2)	11 (3.5)	5 (7.1)	0.185
IE characteristic				
Definite IE	252 (66.3)	206 (66.5)	46 (65.7)	0.906
Possible IE	128 (33.7)	104 (33.5)	24 (34.3)	
Complication at diagnosis	229 (60.3)	173 (55.8)	56 (80.0)	< 0.001
Congestive heart failure	145 (38.2)	111 (35.8)	34 (48.6)	0.048
Arrhythmia	8 (2.1)	4 (1.3)	4 (5.7)	0.033
Neurological	67 (17.6)	47 (15.2)	20 (28.6)	0.009
Other	46 (12.1)	33 (10.6)	13 (18.6)	0.070
Abnormal echocardiogram^a^	324/372 (87.1)	269/308 (87.3)	55/64 (85.9)	0.76
Affected valve				
Mitral valve	166 (51.1)	139 (51.5)	27 (49.1)	0.747
Non-mitral valve	159 (48.9)	131 (48.5)	28 (50.9)	
Vegetation observed^b^	287/372 (77.2)	239/308 (77.6)	48/64 (75.0)	0.653
Size ≥1 cm	118 (35.6)	99 (36.7)	19 (31.1)	0.16
Multiple vegetations	41 (12.4)	14 (4.5)	8 (13.1)	0.58
Inappropriate empirical ABT	57 (15.1)	39 (12.7)	18 (25.7)	0.007
Positive hemoculture^c^	311 (82.3)	251 (81)	60 (87.0)	0.26
*Streptococcus* spp.	189 (60.8)	167 (66.5)	22 (36.7)	
Non-streptococcus	122 (39.2)	84 (33.5)	38 (63.3)	< 0.001
*Staphylococcus* spp.	69 (22.2)	47 (18.7)	22 (36.7)	
Non-staphylococcus	242 (77.8)	204 (81.3)	38 (63.3)	0.002
Surgical treatment	187 (49.2)	168 (54.2)	19 (27.1)	< 0.001
Complication during admission	229 (60.3)	159 (51.3)	70 (100)	
Neurological	44 (11.6)	20 (6.5)	24 (34.3)	< 0.001
Congestive heart failure	103 (27.1)	63 (20.3)	40 (57.1)	< 0.001
Arrhythmia	60 (15.8)	37 (11.9)	23 (32.9)	< 0.001
Hospital-acquired infection	90 (23.7)	51 (16.5)	39 (55.7)	< 0.001
Renal complication	99 (26.1)	63 (20.3)	36 (51.4)	< 0.001
Other	71 (18.7)	40 (12.9)	31 (44.3)	< 0.001

A *p*-value< 0.05 indicates statistical significance

^a^Available data for echocardiography 372 cases

^b^Available data 331 cases

^c^Available data 379 cases

Table 3 shows multivariate analysis for factors associated with mortality. The identified independent risk factors were ESRD (adjusted odds ratio [aOR]: 9.43, 95% confidence interval [CI]: 2.36–37.70); DM (aOR: 2.81, 95% CI: 1.06–7.49); neurological complication on admission (aOR: 14.16, 95% CI: 5.11–39.22); and, complication during admission, including congestive heart failure (aOR: 4.32, 95% CI: 1.91–9.75), hospital-acquired infection (aOR: 3.78, 95% CI: 1.66–8.57), renal complication (aOR: 3.12, 95% CI: 1.32–7.37), and other complication (e.g., septic shock, ARDS, cardiac tamponade, and myocarditis) (aOR: 3.28, 95% CI: 1.41–7.61). Higher platelet level (aOR: 0.51, 95% CI: 0.31–0.84) and surgical intervention (aOR: 0.34, 95% CI: 0.13–0.88) were found to be protective factors against mortality. We also analyzed the data by excluding 62 patients admitted to the surgical department for elective valve repair or replacement to reduce some bias. Among the 318 patients admitted to the medical department, surgery was indicated and performed in 90 patients, and surgery was indicated but not performed in 45 patients. After performing multivariate analysis with 318 participants, surgery remained a protective factor against mortality with an adjusted OR [aOR] of 0.19 (95% CI: 0.07–0.58). The following factors remained significantly associated with mortality: ESRD (aOR: 7.14, 95% CI: 1.42–35.95); DM (aOR: 2.78, 95% CI: 1.02–7.62); neurological complication on admission (aOR: 2.94, 95% CI: 1.09–7.88); and, complication during admission, including congestive heart failure (aOR: 7.76, 95% CI: 3.16–19.10), hospital-acquired infection (aOR: 3.22, 95% CI: 1.38–7.53), renal complication (aOR: 3.61, 95% CI: 1.50–8.68), or other complication (aOR: 3.86, 95% CI: 1.59–9.35). Higher platelet level remained a protective factor against mortality with an aOR of 0.51 (95% CI: 0.31–0.85). In addition, inappropriate antibiotic therapy was found to be independently associated with mortality (aOR: 2.87, 95% CI: 1.03–8.02), whereas IE due to Streptococci was identified as a protective factor against mortality (aOR: 0.17, 95% CI: 0.04–0.83).

**Table 3:** Multivariate analysis for factors associated with overall mortality

Factors	Crude OR (95% CI)	*p*-value	Adjusted OR (95% CI)	*p*-value
Comorbidity				
End-stage renal disease	4.53 (1.84–11.14)	0.001	9.43 (2.36–37.70)	0.002
Diabetes mellitus	3.97 (2.15–7.33)	< 0.001	2.81 (1.06–7.49)	0.039
Complication at diagnosis				
Neurological complication	2.24 (1.22–4.096)	0.009	2.39 (0.92–6.20)	0.074
Complication during admission				
Neurological complication	7.57 (3.87–14.78)	< 0.001	14.16 (5.11–39.22)	< 0.001
Congestive heart failure	5.23 (3.02–9.05)	< 0.001	4.32 (1.91–9.75)	< 0.001
Hospital-acquired infection	6.39 (3.65–11.18)	< 0.001	3.78 (1.66–8.57)	0.002
Renal complication	4.15 (2.41–7.15)	< 0.001	3.12 (1.32–7.37)	0.009
Other	5.37 (3.01–9.55)	< 0.001	3.28 (1.41–7.61)	0.006
Surgical treatment	0.32 (0.18–0.56)	< 0.001	0.34 (0.13–0.88)	0.026
Log platelet	0.43 (0.30–0.60)	< 0.001	0.51 (0.31–0.84)	0.008

A *p*-value< 0.05 indicates statistical significance

^a^Variable(s) entered on step 1: Age, Sex, Type of heart involvement: PVE + DRIE, Presumed mode of acquisition of IE: Healthcare associated, Comorbidity: HT, DM, Hyperlipidemia, ESRD, Inappropriate empirical antibiotic therapy, Hemoculture: non-streptococci, Complication at diagnosis: CHF, arrhythmia, neurological complication, Surgical treatment, Lab parameter: Log platelet, Cr, Complication during admit: Neurological complication, CHF, co-infection, other

^b^Backward stepwise (Likelihood ratio) analysis

^c^Missing cases 72 (18.9%)

## Discussion

This study revealed a higher cumulative incidence of IE than the rates reported from two previous studies conducted in Thailand. The present study found an incidence of 5.67 per 1000 admissions during the 2005–2015 study period, which is higher than the previously reported 2.6 cases per 1000 admissions during 1982–1989 [8], and the previously reported 4 cases per 1000 admissions during 1990–1999 [9]. Moreover, the incidence of IE appears to vary by continent. A systematic review of studies from Europe and the United States during 1970 to 2000 reported an estimated incidence of IE of 1.5 to 6 cases per 100,000 person-years [10]. The incidence of IE in the United States appeared to increase from 11.4 per 100,000 person-years in 1999 to 16.6 per 100,000 person-years in 2006 [11]. Similarly, the incidence increased from 4.1 per 100,000 person-years in 2000–2002 to 4.9 per 100,000 person-years in 2006–2008 in Northeastern Italy [11]. However, no significant change in the incidence of IE over time was observed in Australia [12] or France [13].

The mean age of patients in our cohort was 53 years, which is lower than the mean age of patients in the International Collaboration on Endocarditis-Prospective Cohort Study (ICE-PCS) (57.9 years). Native valve endocarditis (NVE) was the predominant valve type observed in our study, accounting for 81.3% of IE cases. However, the percentage of PVE and DRIE was markedly increased compared with previous study in Thailand, from 5% in 1990–1999 [9] to 18.7% in this study. A similar trend was observed in a recent systematic review of 142 hospital-based studies, which showed an increasing incidence of IE on prosthetic valve from 8.4% in the 1960s to 22.9% in the 2000s [1]. Approximately 40% of PVE and DRIE in our study were healthcare-associated infection. The patients with PVE or DRIE tended to be older, to have more comorbidities, and to be more likely to be nosocomial or non-nosocomial acquisition than NVE. The increase in the proportion of PVE or DRIE is thought to be partly due to the increasing use of invasive medical devices, which has resulted in a change in the pattern of pathogens that cause IE.

VGS remains the most common pathogen group identified in NVE (40%), whereas MSSA and VGS were found with equal frequency (20%) in PVE or DRIE patients. In contrast to our findings, previous study reported a decline in the frequency of IE caused by VGS over time from 27.4% in the 1960s to 17.6% in the 2000s on all continents including Asia [1], and an increased proportion of IE due to *S. aureus* from less than 10% to more than 25% [2, 14]. However, epidemiological studies of IE in Asian populations are scarce. A previous study in 180 IE cases conducted at a large tertiary-care teaching hospital in Japan during 2000 to 2014 [15] found *S. aureus* to be the leading cause of IE (27%), followed by VGS (22%). Of note, the mean age of patients in that study was 69 years, which is far higher than the mean age of patients in other studies. A recent systematic review [4] that included 105 studies from 36 countries (a total of 33,214 IE cases) reported a trend toward more *S. aureus* etiology causing IE, especially among intravenous drug use patients. However, VGS remains the most common pathogen in the sub-group population from Asia. Previous studies conducted at a university hospital in Northeastern Thailand also found VGS to be the predominant pathogen. Nevertheless, it must be noted that 25% of IE cases in one of those two studies from Thailand were found to be caused by zoonotic bacteria [6]. This supports the variability of IE etiology among countries, regions, and continents, which means that a global one-size-fits-all approach to the management of suspected IE is not appropriate. Consistent with other studies, *S. aureus*, CoNS, and *Enterococcus* spp. were the common pathogens found to cause PVE and DRIE [1, 4, 16]. In the present study, selecting ceftriaxone as an empiric antibiotic will cover up to 80% of pathogens that cause NVE.

The in-hospital mortality rate due to IE in our study was 18.4%, which is high, but shows a decline from the 25% rate reported from a previous study conducted in Northeastern Thailand a decade ago [9]. However, the mortality rate in our study was slightly higher than that reported from a previous study that was conducted at our hospital over the past two decades (14%) [8]. It must be noted that the patients in the present study were markedly older and had more comorbid disease than the patients in that previous study [8]. There also appears to be regional variability in patient mortality. Recent studies from Sweden and Finland reported 30-day mortality rates ranging from 10 to 11% [17, 18]. The mortality rate was higher in some developing countries [19–21], ranging from 27 to 32%. The hospital mortality rate in our study was quite similar to the rates reported in a previous systematic review, which showed that the mortality rate remained stable after the 1960s at around 20% [1]. We found the mortality rate to be higher among the PVE or DRIE group compared to the NVE group. This could be due to the fact that the PVE or DRIE groups had more older aged patients, those patients had more comorbidities, and there was more healthcare-associated infection causing more infection with non-streptococci. This may have resulted in a higher percentage of patients receiving inappropriate empirical antibiotic therapy, which could have led to delayed treatment response, more complications, and a higher mortality rate.

ESRD, DM, and occurrence of complications during admission, including neurological complications, congestive heart failure, arrhythmia, hospital-acquired infection, or renal complication showed significant association with mortality in our study. Of interest, surgical treatment and a higher platelet level were shown to be protective factors against mortality. Our findings are somewhat similar to those from a study conducted in Turkey that found being on dialysis, having CNS emboli, and having CHF to be risk factors for mortality; whereas, surgical intervention and higher platelet level were the protective factors against mortality in IE patients [21]. Of note, 16% of our patients were referred and admitted to the CVT unit/ward for elective surgery. As such, most of those patients had already completed antibiotic treatments, which resulted in less severe cases and lower mortality among surgical patients. This may partly explain why surgical treatment was shown to be a protective factor for IE in our study.

This study has some limitations. First, our study’s retrospective design renders it vulnerable to missing or incomplete data. For example, some baseline clinical characteristics were not recorded, particularly among patients transferred and admitted for surgery. In addition, we did not include only primary diagnosis of IE in this study. We have no specific data available on the number of cases that had IE as primary diagnosis. Second, the data included in this study was from a single center only. Third, our center is a national tertiary referral hospital that is commonly referred cases that cannot be effectively managed in other care settings. It is, therefore, possible that our data may not reflect IE incidence and patient characteristics at other centers. For example, a significant proportion of patients had severe disease with complication at time of diagnosis and during admission; thus, our patient population may not precisely reflect the demographic and clinical profile of patients at other hospitals. In addition, we included only I33.0 code to identify diagnosis of IE which may lead to significant bias in estimation of occurrence of IE and may not be fully comparable with previous reports that used other codes such as I38 or I39. Further prospective multicenter studies should be performed to determine the nationwide etiology and mortality of IE in Thailand.

## Conclusions

The incidence of infective endocarditis is increasing in Thailand. PVE or DRIE has a higher mortality rate than NVE. Several factors, including comorbidities and complications that develop during the evolution of IE, were found to be independently associated with in-hospital mortality.

## Data Availability

The datasets used and/or analysed during the current study are available from the corresponding author on reasonable request.

## Abbreviations

ABT: Antibiotic therapy
CA-IE: Community-acquired infective endocarditis
CI: Confidence interval
CoNS: Coagulase-negative staphylococci
CVT: Cardiovascular and thoracic
DM: Diabetes mellitus
DRIE: Device-related infective endocarditis
ESRD: End-stage renal disease
HACEK: *Haemophilus* spp., *Aggregatibacter* spp., *Cardiobacterium* spp*., Eikenella* spp*., Kingella* spp.
HA-IE: Healthcare-associated infective endocarditis
IE: Infective endocarditis
MSSA: Methicillin-sensitive *Staphylococcus aureus*
NIE: Nosocomial infective endocarditis
NNIE: Non-nosocomial infective endocarditis
NVE: Native valve endocarditis
OR: Odds ratio
PVE: Prosthetic valve endocarditis
SD: Standard deviation
TEE: Transesophageal echocardiography
TTE: Transthoracic echocardiography
VGS: Viridans group streptococci
